# The small non-coding RNA sRNA102 regulates *Pseudomonas aeruginosa* virulence and host immunity by targeting the T3SS component PcrG

**DOI:** 10.3389/fcimb.2026.1675468

**Published:** 2026-02-11

**Authors:** Yushan Chen, Bo Wu, Lunhao Yang, Lan Wang, Lin Xiang, Peng Zhou, Yanmei Liu, Song Li, Qiwei Li

**Affiliations:** 1 Department of Laboratory Medicine, The Second Affiliated Hospital of Guangzhou University of Chinese Medicine, Guangzhou, Guangdong, China; 2 Department of Transfusion, The Fourth Affiliated Hospital of Guangzhou Medical University, Guangzhou, Guangdong, China; 3 Department of Clinical Laboratory, The Affiliated Qingyuan Hospital (Qingyuan People’s Hospital), Guangzhou Medical University, Qingyuan, Guangdong, China

**Keywords:** infection, *P. aeruginosa*, pcrG, sRNA102, virulence

## Abstract

This study investigates the regulatory role of a functionally under-characterized small non-coding RNA (sRNA), sRNA102, in *Pseudomonas aeruginosa*, focusing on its mechanisms of influencing bacterial virulence and host immune modulation. Using an *in vivo* murine intraperitoneal infection model and transcriptomic sequencing, we found that the expression of sRNA102 is host immune-dependent: its expression was significantly upregulated by approximately 3-fold in immunocompetent mouse infection models, whereas no significant upregulation was observed in immunodeficient mouse models. This trend was further validated in *in vitro* PAO1-immune cell co-culture systems and a whole-blood infection model. Functional studies demonstrated that sRNA102 enhances hemolytic activity by 2.5-fold, reduces cytotoxicity toward A549 epithelial cells by approximately 43%, and increases adhesion and invasion capabilities by 2.1-fold. Mechanistically, we confirmed that sRNA102 directly targets and positively regulates pcrG, a key gene of the type III secretion system (T3SS), thereby upregulating the expression of the downstream effector protein ExoS. In infection models, the expression of sRNA102 suppressed the expression of the anti-inflammatory cytokine interleukin-10 (IL-10) (downregulated by approximately 50% in the sRNA102-overexpressing infection group), while promoting the expression of the pro-inflammatory cytokine tumor necrosis factor-alpha (TNF-α) and the immunomodulatory marker arginase 1 (Arg1), ultimately leading to increased bacterial colonization in the mouse peritoneal cavity. These findings reveal a novel regulatory pathway driven by sRNA102, which integrates the pcrG-exoS-T3SS axis to modulate bacterial virulence and host immune responses, deepening our understanding of the fine-tuned, sRNA-mediated regulatory mechanisms during *P. aeruginosa* infection.

## 1 Introduction

Bacterial infections were implicated in 1/8 of deaths worldwide and were the second leading cause of mortality in 2019 (Antimicrobial Resistance Collaborators, 2022). Examples include *Pseudomonas aeruginosa* (*P. aeruginosa*), a Gram-negative opportunistic pathogen with high pathogenicity in immunocompromised populations, such as neonates, the elderly, cancer patients, and individuals with cystic fibrosis, severe burns/trauma, immunodeficiency disorders, and other underlying comorbidities (Qin et al., 2022). Owing to its high prevalence and extensive resistance to drugs, *P. aeruginosa* imposes a substantial strain on global healthcare systems. Consequently, the World Health Organization (WHO) designated it as one of the most critical bacterial threats to human life and listed it as a priority pathogen for novel antibiotic development (World Health Organization). Therefore, the development of novel antimicrobial strategies distinct from traditional antibiotics would be imperative for improved patient outcomes. In this regard, it is also noteworthy that anti-virulence strategies represent a promising alternative to conventional antibiotic treatments for bacterial infections (Naga et al., 2023).

The multiple virulence factors in *P. aeruginosa*, including flagella, pili, Lipopolysaccharides (LPS), secretion systems, proteases, quorum sensing, and biofilm formation, could facilitate interbacterial communication and confer drug resistance. Mechanistically, these virulence mechanisms enable *P. aeruginosa* to infect hosts; promote bacterial adhesion, colonization, and invasion; disrupt host tissue integrity; and suppress or evade immune responses; ultimately leading to clinical manifestations (Liao et al., 2022). According to reports, *P. aeruginosa* has six secretion systems (Type I-VI Secretion Systems, T1SS-T6SS), which coordinate host colonization, adhesion, motility, and chemotaxis, thus enhancing its adaptability and pathogenicity in complex environments (Anantharajah et al., 2016). Among these, the type III secretion system (T3SS) plays a particularly critical role in acute infections. It directly “injects” effector proteins (such as ExoS, ExoT, ExoU, and ExoY) into the host cytoplasm, disrupting cellular functions and modulating immune responses (Hauser, 2009; Diaz et al., 2011). The function of T3SS is governed by a sophisticated regulatory network. PcrG, a key cytoplasmic protein within this system, is not only a structural component of the T3SS apparatus but also acts as a cytoplasmic negative feedback regulator. In addition to controlling T3SS secretion activity and specificity, PcrG upregulates the expression of ExoS, playing a significant role in virulence expression (Sundin et al., 2004; Lee et al., 2014). Furthermore, *P. aeruginosa* can leverage the secretion systems to deliver virulence factors into host cells, facilitating immune evasion and bacterial colonization via host immune response modulation (Sharma et al., 2017). Therefore, unraveling the aforementioned virulence regulation processes could crucially enhance our understanding of *P.aeruginosa* pathogenesis.

In addition to protein-coding genes, small non-coding RNAs (sRNAs) play a rapid and precise role in post-transcriptional regulation, contributing to bacterial environmental adaptation and virulence control. Small non-coding regulatory RNAs (sRNAs), typically 50–400 nucleotides in length, play a pivotal role in *P. aeruginosa’s* pathogenicity and virulence mechanisms, including quorum sensing, ion metabolism, biofilm formation, stress responses, host cell invasion, and growth condition adaptation (Nitzan et al., 2017; Pita et al., 2018). These non-coding regulatory elements often exert rapid regulatory effects through post-transcriptional mechanisms, primarily via sRNA-mRNA interactions, which could lead to translational activation or repression (Kavita et al., 2018). Numerous potential intergenic sRNAs have been identified in *P. aeruginosa* through RNA sequencing (RNA-Seq) (Gómez-Lozano et al., 2012; Wurtzel et al., 2012). However, the functional characterization of these novel sRNAs remains unclear. Currently, the CsrA/RsmA system is the most extensively studied sRNA regulatory system in *P. aeruginosa*. The RsmA protein acts as a global post-transcriptional repressor, inhibiting the translation of various acute infection-related genes—including T3SS genes—by binding to the 5’ untranslated region (UTR) of target mRNAs. In contrast, sRNAs such as RsmY and RsmZ function as “molecular sponges” that sequester the RsmA protein, thereby relieving its repression of target genes and facilitating the transition of the bacterium toward a chronic infection phenotype (Chen et al., 2016). We previously established that the sRNA PrrH directly represses LasI, thereby regulating the modulation of virulence factors, biofilm formation, and motility (Lu et al., 2019). These examples underscore the central role of sRNAs in bacterial environmental adaptation and pathogenesis. However, the functions of most sRNAs in *P. aeruginosa* remain largely underexplored, necessitating further research.

Based on this background, the present study aims to identify and characterize functional small non-coding RNAs (sRNAs) that play crucial roles during *P. aeruginosa* infection. We first established a simple and reliable murine intraperitoneal infection model, which facilitates the collection of sufficient bacterial biomass for molecular analysis at the early stages of infection. By comparing the bacterial transcriptomes under *in vivo* infection and *in vitro* culture conditions using RNA sequencing, we identified a significantly upregulated sRNA, sRNA102, whose expression is dependent on the host immune status. This study provides the first functional characterization of sRNA102 during infection. Mechanistically, we demonstrated that sRNA102 directly targets and positively regulates pcrG, a key gene of the type III secretion system (T3SS), thereby upregulating the expression of the effector protein ExoS, thus integrating the pcrG-exoS-T3SS regulatory axis. Further functional analyses revealed that sRNA102 modulates virulence phenotypes of *P. aeruginosa*, including cytotoxicity and invasiveness. At the host level, sRNA102 shapes a microenvironment conducive to bacterial colonization and proliferation by regulating key cytokines (e.g., suppressing IL-10 and promoting TNF-α) and altering macrophage immune status. Collectively, these effects ultimately promote infection. These findings not only uncover a novel virulence regulatory pathway but also offer a potential new target for antivirulence therapy against *P. aeruginosa* infections.

## 2 Materials and methods

### 2.1 Bacterial strains and plasmids

Herein, two bacterial strains were used: *P. aeruginosa* wild-type PAO1 and *E. coli* DH5α, both maintained in our laboratory. The overexpression plasmids (pROp200 and pSTV28), the homologous recombination plasmid (pGSM-MR), and the green fluorescent protein reporter system vector (pUCP30T-*gfp)*were also preserved in our laboratory. Furthermore, *E. coli* DH5α and SM10λπ were prepared as competent cells for constructing overexpression and knockout strains. Unless otherwise specified, all bacterial cultures were incubated at 37°C in an LB medium or on LB agar plates containing 1.5% agar. Antibiotics were appropriately added at the following concentrations: 30 µg/mL Gentamicin (Gm), 16 µg/mL Chloramphenicol (Cm), 100 μg/mL Ampicillin (AMP), and 20 mg/mL polymyxin B.

### 2.2 Cell lines and experimental animals

Two cell lines were used in this study: The murine monocyte-macrophage leukemia cell line RAW264.7 and the human lung adenocarcinoma alveolar basal epithelial cell line A549 (both from Hunan Fenghui Biotechnology Co., Ltd). The RAW264.7 cells were cultured in a high-glucose DMEM medium supplemented with 15% Fetal Bovine Serum (FBS), while A549 cells were maintained in a high-glucose DMEM medium supplemented with 10% FBS. Animal experiments were performed using C57BL/6JGpt mice (Guangdong GemPharmatech Co., Ltd.) and NSG (NOD Scid Gamma) mice [SpePharm (Beijing) Biotechnology Co., Ltd].

### 2.3 Model construction

For the PAO1 *in vitro* model, bacterial cultures were first collected from secondary enrichment at 6 h, followed by RNA extraction and sequencing analysis. For the murine intraperitoneal infection model, a PAO1 bacterial suspension (500 µL; 5×10^9^ CFU/mL in the logarithmic growth phase) was first injected intraperitoneally into C57BL/6JGpt and immunodeficient NSG mice, respectively. At 6 hours post-infection, the peritoneal cavity was lavaged with 2 mL of sterile PBS to collect peritoneal fluid. After centrifugation, the pellet was used for bacterial RNA extraction, while the supernatant was reserved for subsequent analysis. This model was selected due to its high standardization, reproducibility, and efficiency in obtaining sufficient bacterial biomass during the acute infection phase for downstream molecular mechanistic studies. Transcriptome sequencing (RNA-seq) was performed on both the intraperitoneal infection model and *in vitro*-cultured PAO1. **Table 1** shows the sequencing data.

**Table 1:** sRNA102 and annotated RNA-seq data.

Gene name	Gene id	PAO1 6h	C57 6h	NSG 6h	Gene biotype	Gene description
–	sRNA102	27.90211	83.09177	31.29210	non-coding RNA	regulate virulence and host infection
rsmY	PA0527.1	218.244	128.6831	103.9075	non-coding RNA	regulatory RNA RsmY
phrD	PA0714.1	179.5291	154.2952	148.1731	non-coding RNA	PhrD
PA0836.1	PA0836.1	155.8196	50.2365	62.73113	non-coding RNA	P5
PA0887.1	PA0887.1	253.0843	189.3583	172.9002	non-coding RNA	P7
PA1030.1	PA1030.1	91.64772	40.91461	22.4327	non-coding RNA	P8
PA1112.1	PA1112.1	25.53044	32.53915	36.05546	non-coding RNA	product name confidence: class 1
PA1324.1	PA1324.1	2.65942	0	4.970882	non-coding RNA	P9
PA1781.1	PA1781.1	0.770149	0	0	non-coding RNA	P11
PA2744.1	PA2744.1	62.70633	83.98262	43.95306	non-coding RNA	not available
rgsA	PA2958.1	71.70985	44.78739	34.72048	non-coding RNA	RgsA
PA3304.1	PA3304.1	0	0	0	non-coding RNA	P18
phrS	PA3305.1	3371.296	242.3691	318.1364	non-coding RNA	PhrS
amiL	PA3366.1	28.93449	93.86293	39.76706	non-coding RNA	AmiL
rsmZ	PA3621.1	381.4893	103.5729	150.8406	non-coding RNA	regulatory RNA RsmZ
PA4270.1	PA4270.1	131.5204	557.4889	320.5466	non-coding RNA	P26
PA4272.1	PA4272.1	111.1169	390.0418	195.3325	non-coding RNA	P27
PA4406.1	PA4406.1	149.9407	178.7865	164.7492	non-coding RNA	not available
rnpB	PA4421.1	187891.8	86536.24	57098.64	non-coding RNA	RNA component of RNaseP%2C RnpB
PA4451.1	PA4451.1	13.72604	24.22269	7.69685	non-coding RNA	P35
prrF1	PA4704.1	116.0987	305.8315	251.7702	non-coding RNA	regulatory RNA PrrF1
prrF2	PA4704.2	10.35018	282.8572	227.8545	non-coding RNA	regulatory RNA PrrF2
PA4726.1	PA4726.1	30.69233	46.1621	10.4307	non-coding RNA	P36
crcZ	PA4726.11	78791.82	64769.11	91869.91	non-coding RNA	CrcZ
PA4726.2	PA4726.2	10452.15	11197.59	9122.778	non-coding RNA	P30
PA4758.1	PA4758.1	0	0	0	non-coding RNA	P32
PA5181.1	PA5181.1	28.15386	35.28682	26.31204	non-coding RNA	P34
PA5227	PA5227	326.8976	366.5124	532.2473	non-coding RNA	hypothetical protein; PF05164: cell division protein ZapA

### 2.4 Construction of overexpression plasmids and strains

The experimental procedures were as outlined in Zeng S et al (Zeng et al., 2023). Briefly, the sRNA102 and PcrG sequences were first cloned into the *EcoRI* site of pROp200 using a Ready-to-Use Seamless Cloning Kit to obtain the expression vectors pROp200-sRNA102 and pROp200-pcrG. Another sRNA102 expression vector, pSTV28-sRNA102, was generated by cloning the sRNA102 gene into the *PstI/HindIII* sites of pSTV28. Furthermore, to construct the target-*gfp* translational fusion vector pUCP30T-pcrG-*gfp*, GFP’s coding sequence was cloned into the *XbaI/HindIII* sites downstream of the P_lac_ promoter in pUCP30T, generating the reporter vector pUCP30T-*gfp*. A wild-type fragment of PcrG mRNA containing the putative binding sites for sRNA102 was then PCR-amplified and inserted into the *XbaI/NcoI* sites upstream of the first GFP codon in the pUCP30T-*gfp* vector. The reaction solution was transformed into *E. coli* DH5α and selected with Gm or Cm. The overexpression plasmids pROp200 and pROp200-sRNA102 were further transformed into ΔsRNA102 competent cells to construct pROp200-ΔsRNA102 and sRNA102OE-ΔsRNA102 strains for validation in mouse experiments. These overexpression plasmids were verified through direct sequencing and transformed into PAO1 to generate the overexpression strains. **Supplementary Table 1** shows the primer sequences used.

### 2.5 Construction of knockout strains

The procedures were as outlined in Lu Y et al (Lu et al., 2019). A sacB-based suicide vector system was constructed using the homologous recombinant plasmid pGSM. The upstream and downstream recombinant fragments of sRNA102 were then amplified through PCR using the sRNA102-P1, sRNA102-P2, sRNA102-P3, and sRNA102-P4 cloning primers. **Supplementary Table 1** lists the primers used. The two PCR products were gel extracted and then connected via fusion PCR to delete sRNA102. The pGSM plasmid and fusion PCR product were digested using *SacI* and *XbaI*, respectively. After digestion, they were ligated with T4 ligase and transformed into *E. coli* SM10λπ, with selection on Gm. The resulting SM10λπ pGSM-ΔsRNA102 strain was then conjugated with the PAO1 WT strain, with selection on Amp and Gm. The resulting clone, PAO1 pGSM-ΔsRNA102, was reversely screened on a 10% sucrose-LB plate to generate the PAO1 ΔsRNA102 strain, which was transformed with the pROp200 plasmid to construct the pROp200 (ΔsRNA102)-carrying sRNA102-deleted PAO1 strain. **Supplementary Table 1** lists the primer sequences used.

### 2.6 Northern blot

The procedures were as outlined in Bhardwaj AR et al (Bhardwaj et al., 2021). Total RNA was extracted from the vector control, sRNA102-knockout strain (ΔsRNA102), and sRNA102-overexpressing strain (sRNA102OE) using TRIzol Reagent (Beyotime, R0016). Subsequently, sRNA102 expression and molecular size were assessed using Northern blotting. DIA-UP BIOTECH (Beijing, China) synthesized the Biotin-labeled probes. **Supplementary Table 2** lists the sequences of the sRNA102 probe and the 5S rRNA internal control probe, both with 5’-biotin modification. Detection was conducted using a Biotin Northern Blot Kit (for Small RNA) (Beyotime, R0220).

### 2.7 qRT-PCR

First, a single clone was selected from the LB plate of the appropriate strain and grown overnight at 37°C with shaking at 200 rpm in 3 mL LB medium. Subsequently, for the second cultivation, 30 μL culture was added to a new 3 mL LB medium (cell density equivalent) and incubated for the indicated time. Total RNA was extracted and quantified using TRIzol Reagent (Beyotime, R0016) and a NanoDrop spectrophotometer (Thermo Fisher Scientific, USA), respectively. Reverse transcription was performed using a ToloScript All-in-one RT EasyMix for qPCR (TOLOBIO, 22107) with 1 μg of total RNA in a 20 μL reaction volume. Following that, quantitative PCR (qPCR) was conducted in triplicate for each cDNA template using the 2×Q3 SYBR qPCR Master mix (TOLOBIO, 22204) on a CFX96 Touch Real-Time PCR Detection System (Bio-Rad, USA). The Cycle threshold (Ct) values were normalized to the housekeeping gene *rpoD* (for bacterial samples) and *GAPDH* (for mammalian cell samples) using the relative threshold cycle (2^−ΔΔCt^) method. **Supplementary Table 1** lists the qRT-PCR primer sequences.

### 2.8 Model validation

The *P. aeruginosa*-immune cell infection model: First, RAW264.7 cells were seeded evenly into 12-well plates at a density of 1.8×10^6^ cells/well and then incubated overnight in a 37°C, 5% CO_2_ incubator. After cell adhesion, the old medium was aspirated, and the cells were gently washed with PBS buffer. Subsequently, each well was filled with 1 mL of a medium containing PAO1 in the logarithmic growth phase and infected at a Multiplicity of Infection (MOI) of 10:1. The plates were co-cultured in the incubator for 6 h before sample collection.

The *P. aeruginosa* bloodstream infection model: First, bacterial suspensions in the logarithmic growth phase were diluted with physiological saline to 5×10^7^ CFU/mL, and EDTA-anticoagulated whole blood was collected from healthy volunteers. The experiment involved three groups: PAO1, whole blood, and heat-inactivated (56°C for 30 min). For the whole blood and inactivated groups, the diluted bacterial suspension (2.4 mL) was mixed with whole blood (0.6 mL) and co-cultured at 37°C with shaking for 6 h. Following that, the samples were collected and centrifuged at 12,000 rpm for 5 min, after which the supernatant was discarded. The pellets were then washed thrice with PBS, followed by RNA extraction and qPCR analysis to determine the relative expression levels.

### 2.9 Growth curves

Growth curves were assessed as described in Janssen KH et al (Janssen et al., 2020). After subculturing the overnight bacterial culture, the suspensions were standardized to 5×10^7^ CFU/mL with a liquid LB medium. A 200 μL aliquot of each standardized culture was then added to a 96-well plate and incubated in a constant-temperature shaking incubator at 37°C for 16 h. The OD600 value was measured every 30 min using a multimode microplate reader (Thermo Fisher Scientific, USA). Each strain’s growth kinetic curves were plotted with incubation time (h) and OD600 values as the x and y axes, respectively.

### 2.10 Fluorescence intensity measurement

A GFP reporter system was established based on the experimental method described by Pu J et al (Pu et al., 2022a). Briefly, *E. coli* DH5α with pUCP30T-mRNA-*gfp* combined with pSTV28 or pSTV28-sRNA102 was grown overnight (8~10 h) at 37°C and re-incubated to 0.5 McFarland standard (MCF). The cultures (100 μL) were then collected and added into 3 mL of LB, and allowed to grow for 6 h. Subsequently, the bacterial cells were centrifuged at 12000 rpm for 1 min, after which the supernatant was discarded. The resulting pellets were then washed twice with PBS and resuspended. For fluorescence microscopy, the bacterial suspension (5 µL) was evenly spread on a clean glass slide and observed under an Axio Observer 7 inverted fluorescence microscope (Zeiss, Germany) to capture fluorescence intensity images. For fluorescence intensity quantification, the bacterial suspension (200 µL) was added to a black opaque 96-well plate, and both OD595 and fluorescence intensity F485/535 (excitation: 485 nm, emission: 535 nm) were measured using a Synergy H1 Hybrid Multi-Mode Microplate Reader (BioTek, USA). Fluorescence intensity was expressed as F485/535/OD595.

### 2.11 Biofilm formation assays

The biofilm formation capacity of *P. aeruginosa* was assessed as described by Carloni S et al (Carloni et al., 2017). First, the bacterial suspension (5×10^7^ CFU/mL) was incubated in a 12-well plate at 37°C for 24 h. After removing planktonic bacteria, the biofilm was washed with PBS, fixed with methanol, and stained with 1% crystal violet. The biofilm was then washed and dried before being dissolved in 95% ethanol and measuring OD600 using a microplate reader. Crystal violet-stained biofilms were further observed under a microscope with different magnifications.

### 2.12 Pyocyanin assays

The procedures were as described by Essar DW et al (Essar et al., 1990). To measure pyocyanin production, the bacterial cultures (5×10^7^ CFU/mL) were first incubated at 37°C for 24 h. The supernatant was then extracted with chloroform, mixed with 1 N HCl, and centrifuged. Following that, OD_520_ of the aqueous phase was measured, and pyocyanin concentration was calculated as 17.072×OD_520/_OD_600_. On the other hand, relative production was expressed as experimental group/control group.

### 2.13 Rhamnolipid assays

The procedures were as described by Pinzon NM et al (Pinzon and Ju, 2009). To measure rhamnolipid production, 30 µL of logarithmic-phase bacterial culture was first mixed with 3 mL of the M9 medium and incubated at 37°C with shaking at 200 rpm for 8 h. After centrifugation at 12000 rpm for 30 min, the supernatant (1 mL) was collected and acidified to a pH of 2.5 ± 0.2 with 1 N HCl. The acidified supernatant was extracted with chloroform (4 mL), and 3 mL of the chloroform layer was reacted with a freshly prepared methylene blue solution. After mixing and resting for 15 min, the chloroform layer (200 µL) was transferred to a 96-well plate for OD_638_ measurement.

### 2.14 Adhesion/invasion assays and anti-phagocytic activity tests

The procedures were as described by Xiong JZ et al (Xiong et al., 2024). Adhesion assay: A549 cells (MOI = 10:1) were first infected with bacterial cultures at 37°C with 5% CO_2_ for 1 h. After removing the medium and washing with PBS, the cells were resuspended in ddH_2_O and serially diluted (20-fold dilutions). The diluted solutions (5 µL) were plated on nutrient agar and incubated overnight before counting the colonies. Invasion assay: A549 cells (MOI = 10:1) were first infected with bacterial cultures at 37°C with 5% CO_2_ for 1 h. After washing with PBS, 1 mL of polymyxin B (20 µg/mL) in DMEM was added, and the cells were incubated for 2 h. The cells were then washed, resuspended in ddH_2_O, and serially diluted (10-fold dilutions). Subsequently, the diluted solutions (10 µL) were plated on nutrient agar and incubated overnight before counting the colonies. Anti-phagocytic activity was assessed using RAW264.7 cells following a procedure similar to the one described above.

### 2.15 Hemolysis assay

The hemolysis assay was performed following the methods proposed by Pu J et al (Pu et al., 2022b). Briefly, 1.5 ml of human whole blood was centrifuged at 4000 rpm for 5 min, and the bottom RBC layer was carefully aspirated, washed three times with PBS, and resuspended to obtain a 4% RBC suspension. Next, the bacterial strains were cultured and passaged until they reached the logarithmic growth phase, then centrifuged at 12000 rpm for 2 min, washed twice with PBS, and adjusted to 5×10^7^ CFU/ml. 300 μl of 4% RBC suspension was mixed with 300 μl of bacterial suspension and co-cultured at 200 rpm, 37°C for 4 h. In this test, PBS and RBC lysis buffer were used as negative and positive controls, respectively. The mixture was centrifuged 4000 rpm for 3 min, 200 μl of the supernatant was transferred to a 96-well plate, and the absorbance at OD450 was measured using a microplate reader. The hemolysis rate was calculated as (A_co-culture_ - A_negative control_)/(A_positive control_ - A_negative control_) × 100%.

### 2.16 Cell counting kit-8 assay

The A549 cells were seeded onto a 96-well plate at a density of 8×10^3^ cells/well and incubated overnight. Once cell adherence was formed, bacterial suspensions in the logarithmic growth phase were added to each well at a MOI of 10:1. Following a 3-hour incubation, 10 µL of CCK-8 reagent (Beyotime, C0039) was added to each well. The plate was further incubated for 2 h at 37°C, and the absorbance at OD450 was recorded using a microplate reader.

### 2.17 Cytokine analysis

Secondary enriched bacterial cultures in the logarithmic growth phase were used to infect RAW264.7 cells (MOI = 10:1) and A549 cells (MOI = 15:1) in FBS-free medium. Briefly, the cells were co-cultured at 37°C in incubator with 5% CO_2_ for 2 h, and then subjected to the qPCR to quantify the expression of cytokines Interleukin-6 (IL-6), Interleukin-10 (IL-10), Interleukin-1β (IL-1β), and inducible Nitric Oxide Synthase (iNOS).

### 2.18 *In vivo* mouse experiments

C57BL/6JGpt mice were intraperitoneally injected with 200 µL of bacterial suspension (1×10^7^ CFU/mL) or sterile PBS (as a negative control). Two hours later, the mice were euthanized, and peritoneal fluid was collected by rinsing the cavity with 2 mL PBS. Following the method described in the Sundarasivarao PYK et al (Sundarasivarao et al., 2022), a portion was serially diluted for colony-forming unit (CFU) counting on LB agar, and the remainder was subjected to RNA extraction and qRT-PCR analysis.

### 2.19 Statistical analysis

All experiments were performed in triplicate. Data are expressed as the mean ± standard deviation. All statistical tests were performed based on the assumption of a normal distribution (Shapiro-Wilk test). Student’s *t* test or one-way analysis of variance (ANOVA) followed by the *LSD post hoc* test in the SPSS (IBM corp. Version 24, Armonk, NY, USA) statistics software. A *P*-value of less than 0.05 was considered statistically significant, with *P* values considered as *P<0.05; **P<0.01; and ***P<0.001. Graphs were plotted using GraphPad Prism software (Version GraphPad Software 9.1, Boston, MA, USA).

## 3 Results

### 3.1 Identification and characterization of sRNA102 in *P. aeruginosa* infection models

**Figure 1:**
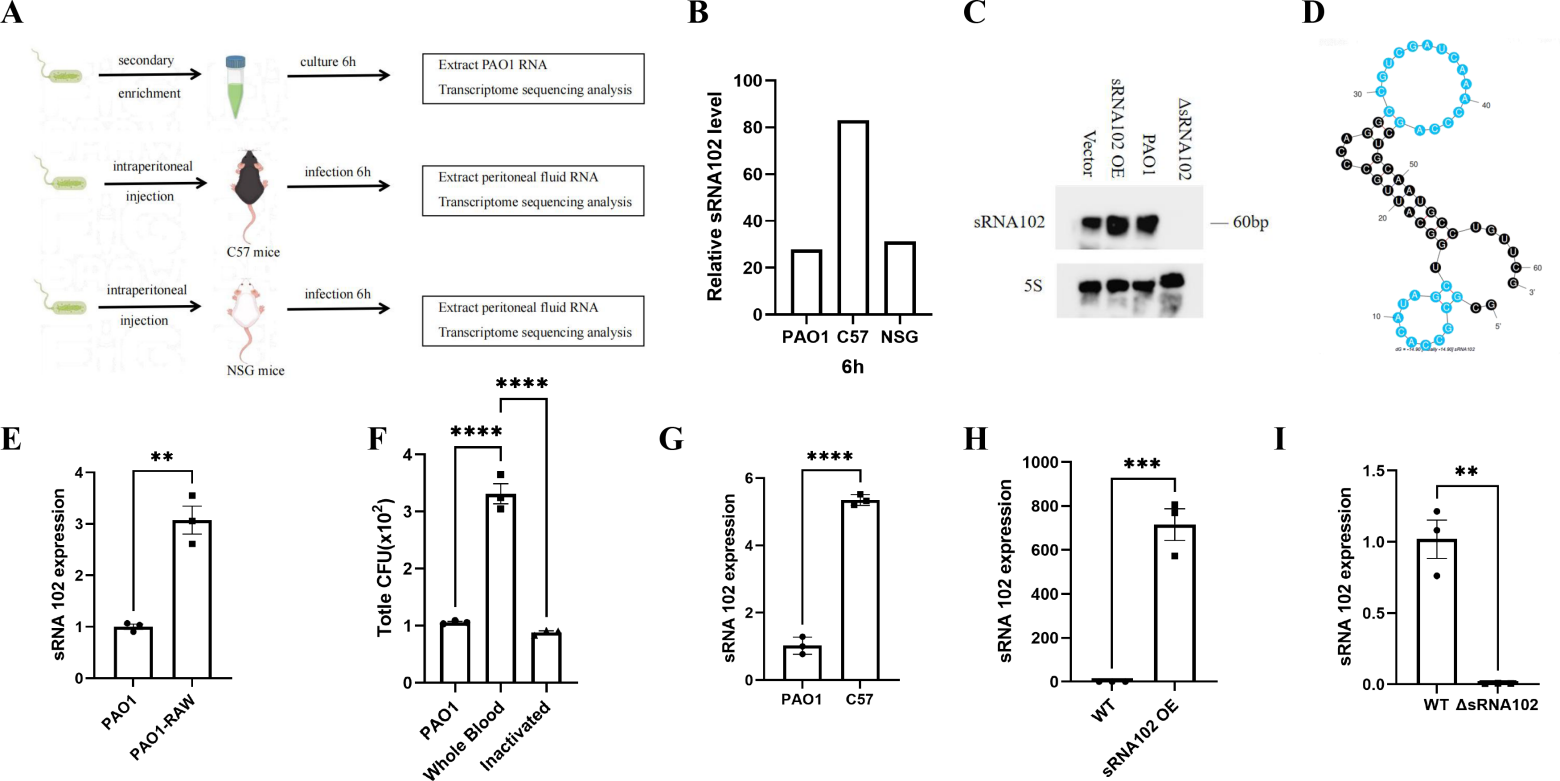
Identification of sRNA102 was confirmed to be a novel small non-coding regulatory RNA, consistent with the expression trends observed in the preliminary model. **(A)** Schematic diagram showing the PAO1 infection models. **(B)** Expression levels of sRNA102 in *in vitro* culture, C57BL/6JGpt, and NSG mouse infection models (RNA-seq data) **(C)** Northern blot validation of non-coding regulatory small RNA102. **(D)** Predicted secondary structure of sRNA102. **(E)** Co-culture model with RAW264.7 cells. **(F)** Bloodstream infection model **(G)** Validation of sRNA102 expression in the C57BL/6JGpt mice *in vivo* intraperitoneal infection model. **(H)** Comparison of sRNA102 expression between overexpressing strain compared to the wild-Type strain. **(I)** Expression of sRNA102 is nearly undetectable in the knockout strain. The data are shown as the mean ± standard error of the mean of at least three independent experiments. **P <0.01; ***P <0.001; ****P <0.0001; ns, non-significant.

To identify sRNAs that play critical roles during infection, we established a murine intraperitoneal infection model (**Figure 1A**).In this study, analysis of the sequencing data from our previously constructed murine intraperitoneal infection model and the *in vitro* PAO1 model revealed that sRNA102 has an important role in infection and immunity. Notably, compared to the *in vitro* culture level, the expression of sRNA102 was significantly upregulated by approximately 3-fold in the C57BL/6JGpt mouse intraperitoneal infection model, whereas this phenomenon was not observed in the immunodeficient NSG mouse model of intraperitoneal infection (**Figure 1B**). Further analysis of Northern blot results from both the wild-type PAO1 strain and the sRNA102-overexpressing strain confirmed that sRNA102 is a novel small non-coding RNA, with a sequence length of 61 nucleotides (located at positions 3329828 to 3329888 on the reference genome NC_002516.2). (**Figure 1C**). The secondary structure of sRNA102 was predicted using the RNA Folding Form website as shown in **Figure 1D**.

To validate the sequencing results, we examined the expression of sRNA102 across multiple models. qPCR analysis showed that after 6 hours of co-culture with RAW264.7 macrophages, the expression of sRNA102 in PAO1 was upregulated approximately 3.1-fold (**Figure 1E**). In a whole-blood infection model, sRNA102 expression was upregulated in the fresh whole blood group but showed no significant change in the heat-inactivated whole blood group (**Figure 1F**). *In vivo* experiments further confirmed that sRNA102 expression was upregulated about 5.2-fold in the C57BL/6JGpt mouse intraperitoneal infection model (**Figure 1G**). These results consistently indicate that sRNA102 expression is strongly induced by the host immune environment.

We successfully constructed sRNA102-overexpressing (sRNA102OE) and knockout (ΔsRNA102) strains, and their expression changes were verified by qPCR (**Figures 1H, I**).

### 3.2 sRNA102 enhances virulence phenotypes of *P. aeruginosa*

**Figure 2:**
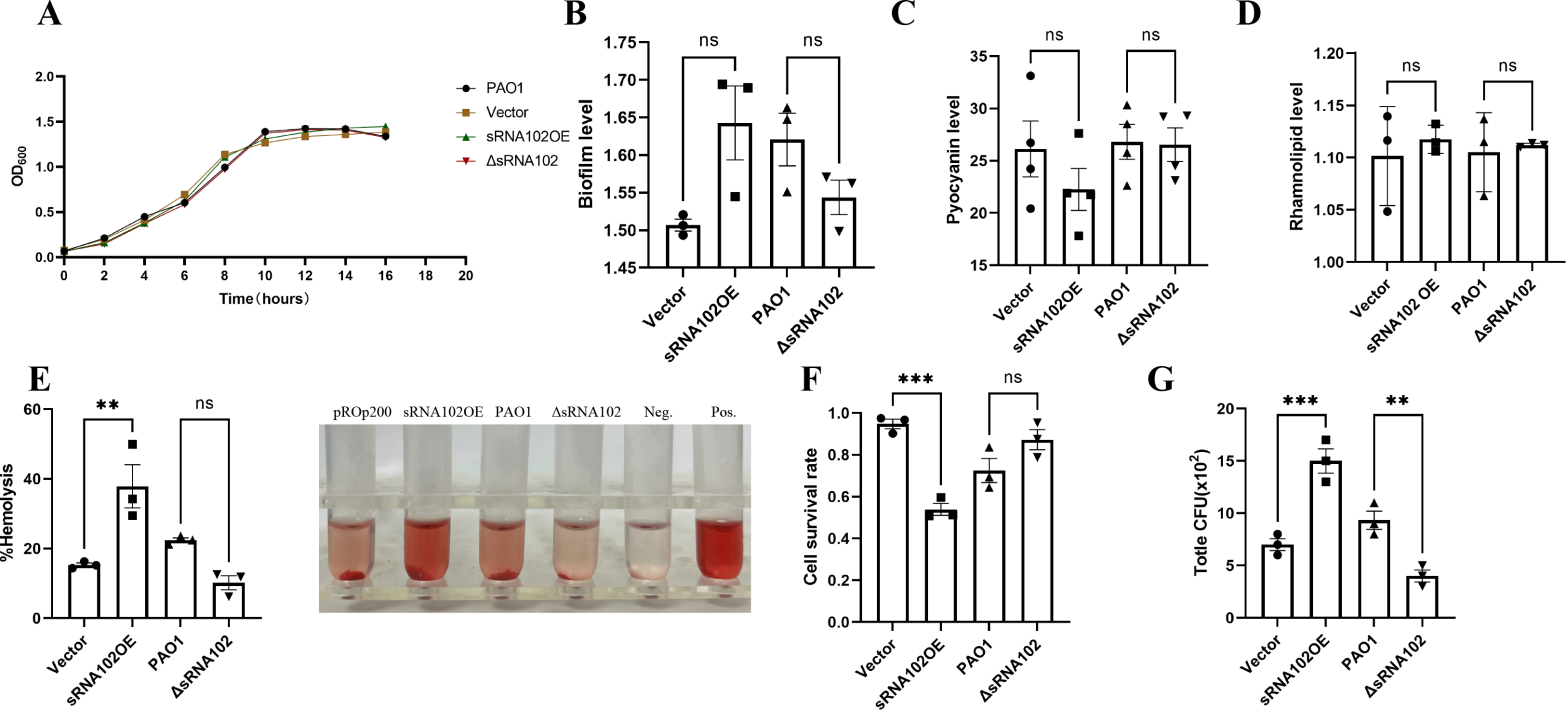
Analysis of the impact of sRNA102 on virulence-related phenotypes. **(A)** Growth curve. **(B)** sRNA102 has no significant effect on biofilm formation. **(C)** sRNA102 does not affect pyocyanin production. **(D)** sRNA102 does not influence rhamnolipid production. **(E)** hemolytic activity Assay. **(F)** sRNA102 reduces cell viability and enhances the toxicity of *P. aeruginosa* to host cells. **(G)** sRNA102 promotes the invasion ability (A549 cells were infected at a MOI of 10:1). The data are shown as the mean ± standard error of the mean of at least three independent experiments. **P <0.01; ***P <0.001; ns, non-significant.

To clarify the role of sRNA102, we investigated its effects on *P. aeruginosa* growth. Notably, there was no significant difference in growth curves of the PAO1 wild-type (PAO1), pROp200-PAO1 (pROp200), sRNA102-overexpression strain (sRNA102OE) and sRNA102-knockout strain (ΔsRNA102) (**Figure 2A**). Furthermore, overexpression of sRNA102 significantly enhanced the hemolytic activity of Pseudomonas aeruginosa, with the hemolysis rate increasing approximately 2.5-fold compared to the empty vector control, while its knockout attenuated this capability (**Figure 2E**). However, sRNA102 overexpression showed no significant effect on biofilm formation, pyocyanin production, or rhamnolipid synthesis (**Figures 2B–D**). Additionally, CCK-8 assay results indicated that the viability of A549 epithelial cells infected with the sRNA102OE strain decreased by approximately 43% compared to the empty vector control, whereas the viability in the ΔsRNA102 group was higher than that in the PAO1 group, suggesting that sRNA102 may stimulate the toxicity of P. aeruginosa toward host cells (**Figure 2F**). The invasion ability of cells from the sRNA102OE group was superior to that of the pROp200 group, while that of the ΔsRNA102 group was significantly weaker than that of the PAO1 group (**Figure 2G**).

Collectively, these results showed that sRNA102 participated in the regulation of *P. aeruginosa* virulence.

### 3.3 PcrG is a direct target for sRNA102 to exert its virulence regulatory function

**Figure 3:**
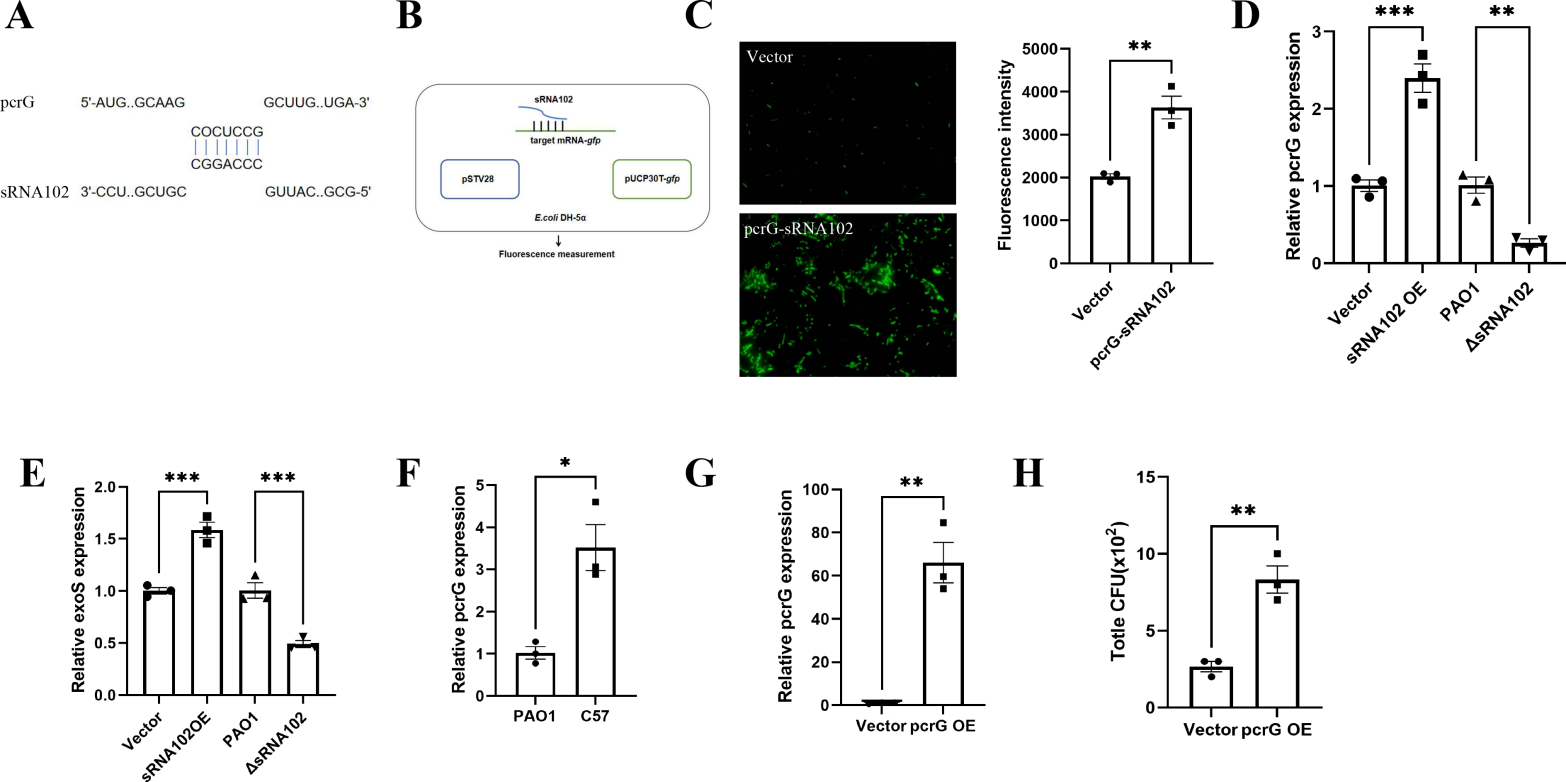
sRNA102 directly targets PcrG and regulates invasive capacity. **(A)** The predicted binding sites between sRNA102 and PcrG. **(B)** A Green Fluorescent Protein (GFP) reporter system indicating the direct interactions of sRNA102 with its potential targets. **(C)** The plasmids pSTV28 (Vector) and pSTV28-sRNA102 (pcrG-sRNA102) were cotransformed with a GFP reporter plasmid (pUCP30T-*gfp*) containing the wild-type sequence of PcrG mRNA into *E. coli* DH5α. The fluorescence was measured using a microscope, and the intensity was detected using a BioTek Synergy H1 microplate reader, expressed in arbitrary units (AU) as F485/535. **(D)** The relative expression level of pcrG in the sRNA102OE and ΔsRNA102 as measured by the qRT-PCR assay. **(E)** The relative expression level of exoS in the sRNA102OE and ΔsRNA102 was detected by qRT-PCR. **(F)** Validation of pcrG expression in the C57BL/6JGpt mice *in vivo* intraperitoneal infection model. **(G)** Validation of the pcrG-overexpressing strain (pcrG OE) by the qPCR test. **(H)** pcrG overexpression promotes the invasive ability in *P. aeruginosa*. The data are shown as the mean ± standard error of the mean of at least three independent experiments. *P <0.05; **P <0.01; ***P <0.001; ns, non-significant.

To identify the molecular mechanisms in which sRNA102 influences the virulence phenotypes, target genes were predicted on the IntaRNA. The PcrG, which is associated with T3SS, was identified as a potential target (**Figure 3A**). In subsequent tests, we developed the green fluorescent protein (GFP) reporter to clarify whether PcrG is a direct target of sRNA102 (**Figure 3B**). It was observed that the fluorescence intensity in sRNA102-overexpression group (pcrG-sRNA102) was significantly enhanced (**Figure 3C**). Similarly, we carried out qRT-PCR, which revealed that the endogenous expression of PcrG was significantly increased in the sRNA102-overexpression strain but decreased in sRNA102-knockout strain (**Figure 3D**). PcrG upregulated the expression of ExoS, a key virulence effector of the T3SS. Based on these results, we further quantified the mRNA level of ExoS through qRT-PCR analysis. This uncovered that the endogenous expression of exoS was significantly upregulated in the strain with sRNA102-overexpressionand decreased in the strain with sRNA102-knockout, which was consistent with the expression trend of pcrG (**Figure 3E**). Notably, in our mouse infection model, the expression pattern of PcrG mirrored that of sRNA102 (**Figure 3F**). These results provide evidence that sRNA102 can bind to the mRNA of PcrG through base-pairing, thereby promoting mRNA translation and enhancing its stability.

Subsequently, we explored the role of PcrG in influencing the invasive ability. based on the developed pcrG-overexpression strain (pcrG OE) (**Figure 3G**), we found that the invasive ability increased relative to the control group (**Figure 3H**). These results showed that sRNA102 promoted invasive ability by targeting PcrG.

### 3.4 sRNA102 assisted *P. aeruginosa* infection by enhancing host immunity

**Figure 4:**
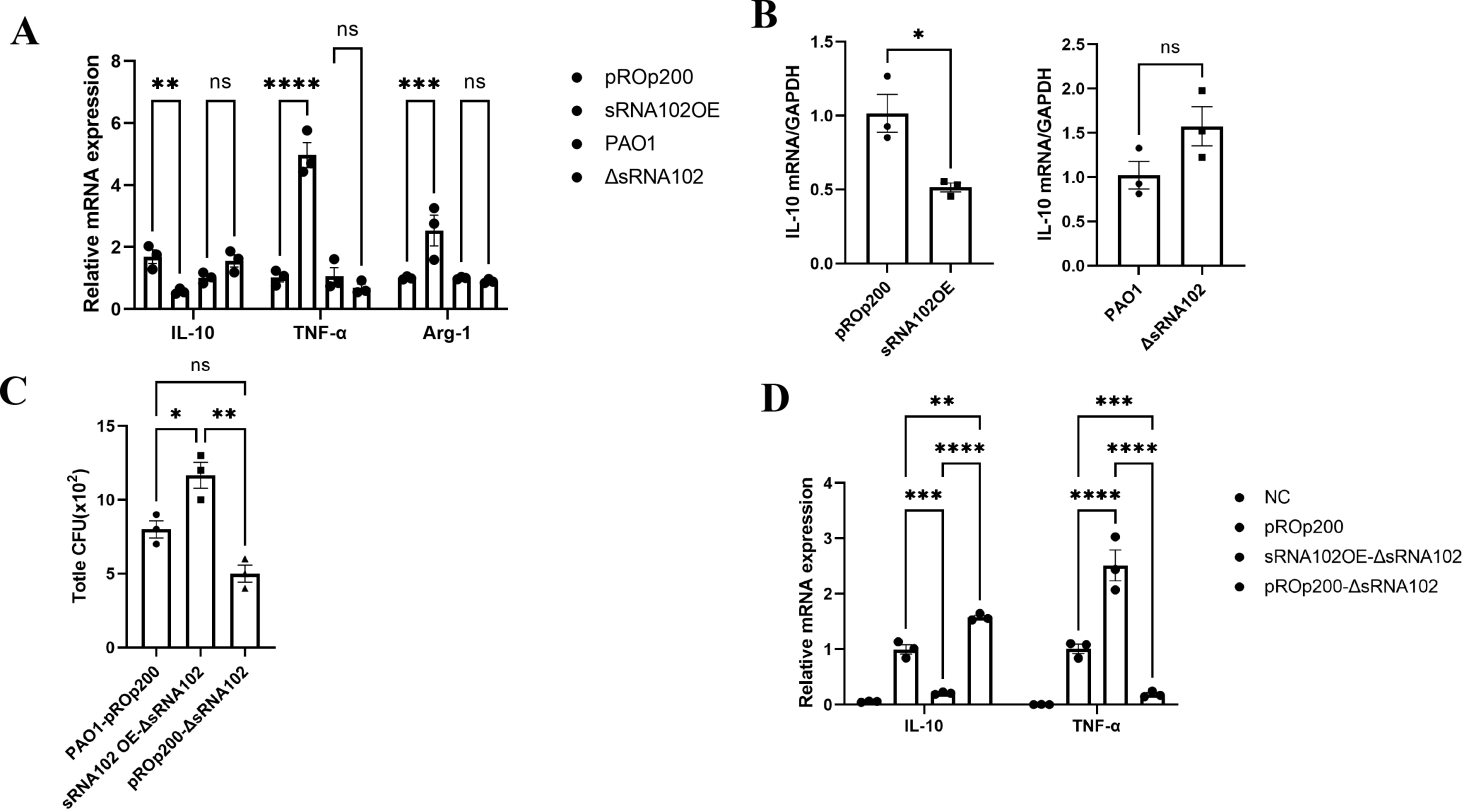
**(A)** The impact of sRNA102 on cytokine expression (RAW264.7 cells). **(B)** Effect of sRNA102 on cytokine expression (A549 cells). **(C)** The CFU counts in mice peritoneal fluid 6 hours post-infection. **(D)** The impact of sRNA102 on the expression levels of inflammatory factors in mouse peritoneal fluid as determined by qRT-PCR. The data are shown as the mean ± standard error of the mean of at least three independent experiments. *P <0.05; **P <0.01; ***P <0.001; ****, *P* <0.0001; ns, non-significant.

We further investigated the impact of sRNA102 on host immune responses. Research has reported that bacterial virulence is influenced by the host immune response. Thus, we quantified the expression level of inflammation-associated cytokines following the co-culture of PAO1 and RAW264.7 cells or A549 for 6 hours, to determine whether sRNA102 could alter the immune function in response to *P. aeruginosa* infection in the host cells. Compared to the control group, RAW264.7 cells infected with the sRNA102-overexpressing strain exhibited elevated levels of TNF-α and the M1-type marker Arg-1, while the expression of the anti-inflammatory factor IL-10 was suppressed by approximately 55%. Furthermore, RAW264.7 cells infected with the sRNA102-knockout strain showed upregulated IL-10 expression (**Figure 4A**). Similarly, in A549 cells, sRNA102 overexpression led to a decrease in IL-10 expression of about 50%, whereas sRNA102 knockout resulted in increased IL-10 expression (**Figure 4B**).

In further tests, we measured the effect of sRNA102 on *P. aeruginosa* infection and host immunity using the mouse peritoneal infection model. Quantification of the bacterial counts revealed that the number of sRNA102 overexpressing strains was higher and sRNA102 knockout strains had reduced counts compared to the controls 6 hours after intraperitoneal infection in mice (**Figure 4C**).

Similar to the results of *in vitro* experiments, sRNA102 overexpression downregulated IL-10 whereas sRNA102 knockout upregulated its expression. Moreover, sRNA102 overexpression upregulated the expression of TNF-α while its knockout suppressed its expression (**Figure 4D**).

Altogether, the aforementioned results suggest that upregulation of sRNA102 expression during infection may help *P. aeruginosa* escape host’s immunity and contribute to infection.

## 4 Discussion

This study identified and characterized the previously unknown regulatory function of a small RNA, sRNA102, during acute infection. We demonstrated that sRNA102 is utilized by *P. aeruginosa* through its targeted regulation of the type III secretion system (T3SS) regulatory gene pcrG.sRNA102 enhances PcrG expression, further upregulating the ExoS expression, which promotes the virulence of *P. aeruginosa*, in terms of hemolytic activity, cytotoxicity, invasive ability, and survival capacity in the host. The increase in sRNA102 expression can improve the capacity of *P. aeruginosa* to coordinate the host immune response during infection, creating favorable conditions for bacterial infection and colonization, further establishing an infection.

*P. aeruginosa* is a common conditionally pathogenic bacterium, known to cause skin and lung infections. Therefore, investigations into the mechanism of infection of *P. aeruginosa* are mostly performed using mouse skin infection model (chronic infection study) and lung infection model (acute infection study) (Raz et al., 2019; Yang et al., 2019; Cao et al., 2023). However, these models do not provide convenient real-time collection of sufficient number of bacteria for gene expression regulation studies. Therefore, we developed a mouse intraperitoneal infection model, from which *P. aeruginosa* were extracted from the intraperitoneal fluid 6 hours post-infection for subsequent transcriptome sequencing. Considering that intraperitoneal infection is a common route of *P. aeruginosa* infection, the developed model is simple, fast and ideal for studying gene expression changes during the infection process. Moreover, studies have shown that *P. aeruginosa* infection may be influenced by the host immune function (Moser et al., 2021; Grandy et al., 2023; Hastings et al., 2023), and thus, we included an immunodeficient mice. Notably, the established model allowed us to not only verify the changes in gene expression of already reported sRNAs such as RsmA and PrrH during *P. aeruginosa* infection (Lu et al., 2019), but also uncover new sRNAs that have not been annotated before. In this study, we demonstrated the virulence and immune-regulatory functions of sRNA102, however, additional functional studies are advocated to identify more sRNAs.

Numerous investigations have demonstrated the function of sRNAs in the bacterial adaptation mechanisms to the environment under acute and chronic infections of *P. aeruginosa*. For example, PrrF, a *P. aeruginosa* sRNA, was reported to be promote PQS production and virulence under low-iron conditions in a murine acute lung infection model (Reinhart et al., 2017). Another sRNA, PqsS, was found to stimulate acute infections while inhibiting biofilm-related chronic infection traits (Jia et al., 2024). In the current infection model, sRNA102 expression exhibited host immune-dependent characteristics. For instance, it was significantly upregulated in immunocompetent mice with infection, but downregulated in immunodeficient mice, suggesting that the host immune signals modulate the expression pattern of sRNA102 in response to infection pressure. *In vitro* cell infection and *in vivo* mouse intraperitoneal infection models revealed that sRNA102 downregulated the expression of the anti-inflammatory cytokine IL-10 and increased that of pro-inflammatory cytokine TNF-α and the M2 macrophage polarization marker Arg1. Through this mechanism, it stimulated the inflammatory response in the host and inhibited anti-inflammatory responses, facilitating immune evasion and creating favorable conditions for *P. aeruginosa* infection and colonization (Belo et al., 2021). It is noteworthy that although this study focuses on the pro-inflammatory function of sRNA102, existing research has reported that sRNAs may possess anti-inflammatory or immunosuppressive functions. For example, Koeppen et al. found that sRNAs released by *P. aeruginosa* could inhibit inflammatory responses through host immune signaling pathways, and Pittaluga et al. also reported that *P. aeruginosa* sRNA could regulate innate immune responses under specific conditions (Pittaluga et al., 2024; Xie et al., 2024), These studies suggest that sRNAs may exhibit functional diversity in host-pathogen interactions, with their specific effects potentially dependent on various factors, such as bacterial strain, infection microenvironment, and host immune status.

*P. aeruginosa* employs the T3SS to inject toxins into the cytoplasm of host cells upon contact. Evidence from functional research indicates that sRNA102 directly targets and promotes the expression of virulence-related gene pcrG via base-pairing mechanism. PcrG is a cytoplasmic negative feedback regulator of the T3SS, which is involved in the regulation of the secretion of effectors via its C-terminal residues (Lee et al., 2010). Functionally, PcrG influences the secretion activity and specificity of the T3SS apparatus, which impacts the entry of effectors into the T3SS channel and their secretion activity (Lee et al., 2014). As a key regulator of the T3SS, pcrG can increase the expression of exoS, which in turn determines bacterial toxin secretion, to activate T3SS. It has been reported that PcrG stimulates the expression of extracellular effector ExoS, which is a T3SS-targeted effector of *P. aeruginosa* that translocates effectors to host cells. Structurally, ExoS is a 48.3 kDa protein that contains 453 amino acids and plays a role in the occurrence of the host cell apoptosis via its GAP region or ADP-ribosyltransferase (ADPr) activity (Kroken et al., 2022). Furthermore, ExoS exhibits ADP-ribosyltransferase (ADPRT) activity, triggering apoptosis in host cells infected with *P. aeruginosa* by targeting multiple Ras proteins (Jia et al., 2006). The expression level of ExoS is increased following cell contact, facilitating the polarized delivery of effectors to target cells (Sundin et al., 2004). The ExoS effector protein produced by T3SS is known to interfere with the actin cytoskeleton and other signaling pathways, causing the host cell death, tissue damage, and inflammatory responses. In addition, T3SS effector proteins can inhibit the phagocytic function of host immune cells (e.g., macrophages and neutrophils) or interfere with immune signaling pathways, which blocks the generation of the innate and adaptive immune responses. In macrophages, ExoS can modulate phagocytic vacuole escape through a mechanism that involves the MgtC and OprF and modulation of GAP activity of ExoS (Garai et al., 2019). In neutrophils, ExoS reduce bacterial killing by blocking the phagocytic NADPHoxidase generating reactive oxygen species (Vareechon et al., 2017). ExoS induces *P. aeruginosa*-afflicted host cell apoptosis and colonization by targeting the JNKS signal pathway (Horna and Ruiz, 2021). Notably, the interaction between PcrG and PcrV in the bacterial cytoplasm influences the secretion of bacterial toxin (Nanao et al., 2003). This enables the bacteria the escape immune clearance and improve their survival within the host.

In this study, results of the experiments demonstrated a regulatory role for sRNA102 on bacterial virulence phenotypes, owing to its direct targeting and enhancement of the expression of the virulence-related gene pcrG via base-pairing. We found that sRNA102 improved the hemolytic activity, cytotoxicity, and host cell invasion, thereby improving bacterial colonization and proliferation efficiency in mice. By targeting pcrG, sRNA102 integrates the pcrG-exoS-T3SS axis pathway to induce the secretion of ExoS. It also controls the host immune response by reducing the production of anti-inflammatory cytokine IL-10 and promoting the synthesis of the pro-inflammatory cytokine TNF-α and the M2 macrophage polarization marker Arg1, leading to increased host inflammatory responses and disruption of immune homeostasis. Therefore, this study unveils novel mechanisms driving the virulence and immune regulation of *P. aeruginosa* infection involving sRNA and post-transcriptional regulation of PcrG, which stimulates bacterial virulence phenotypes and promotes the bacterial survival, proliferation, and immune evasion within the host by modulating the host immune microenvironment.

Although the murine intraperitoneal infection model employed in this study facilitated the acquisition of sufficient bacterial biomass for molecular mechanistic analysis during early infection, it differs in microenvironment from the primary clinical sites of *P. aeruginosa* infection (e.g., respiratory tract, wounds). Firstly, our focus has been primarily on the bacterial regulatory mechanisms, leaving the precise perturbation of downstream host cell signaling pathways by the sRNA102-pcrG-exoS axis insufficiently explored. Secondly, given that the main objective of this study was to discover and preliminarily validate a novel regulatory axis, standardized cell lines (RAW264.7, A549) and the intraperitoneal infection model were utilized. While these models hold significant utility for mechanistic exploration, validation of the findings in more clinically relevant infection models would substantially enhance the physiological and pathological relevance of the conclusions.

In summary, we show that sRNA102 enhances hemolytic activity, cytotoxicity, survival capacity within the host and invasive ability by enhancing the expression level of pcrG. Furthermore, it modulates the immune response by downregulates IL-10 and upregulates TNF-α and Arg1, thereby enhancing *P. aeruginosa* infection of the host.

**Figure 5:**
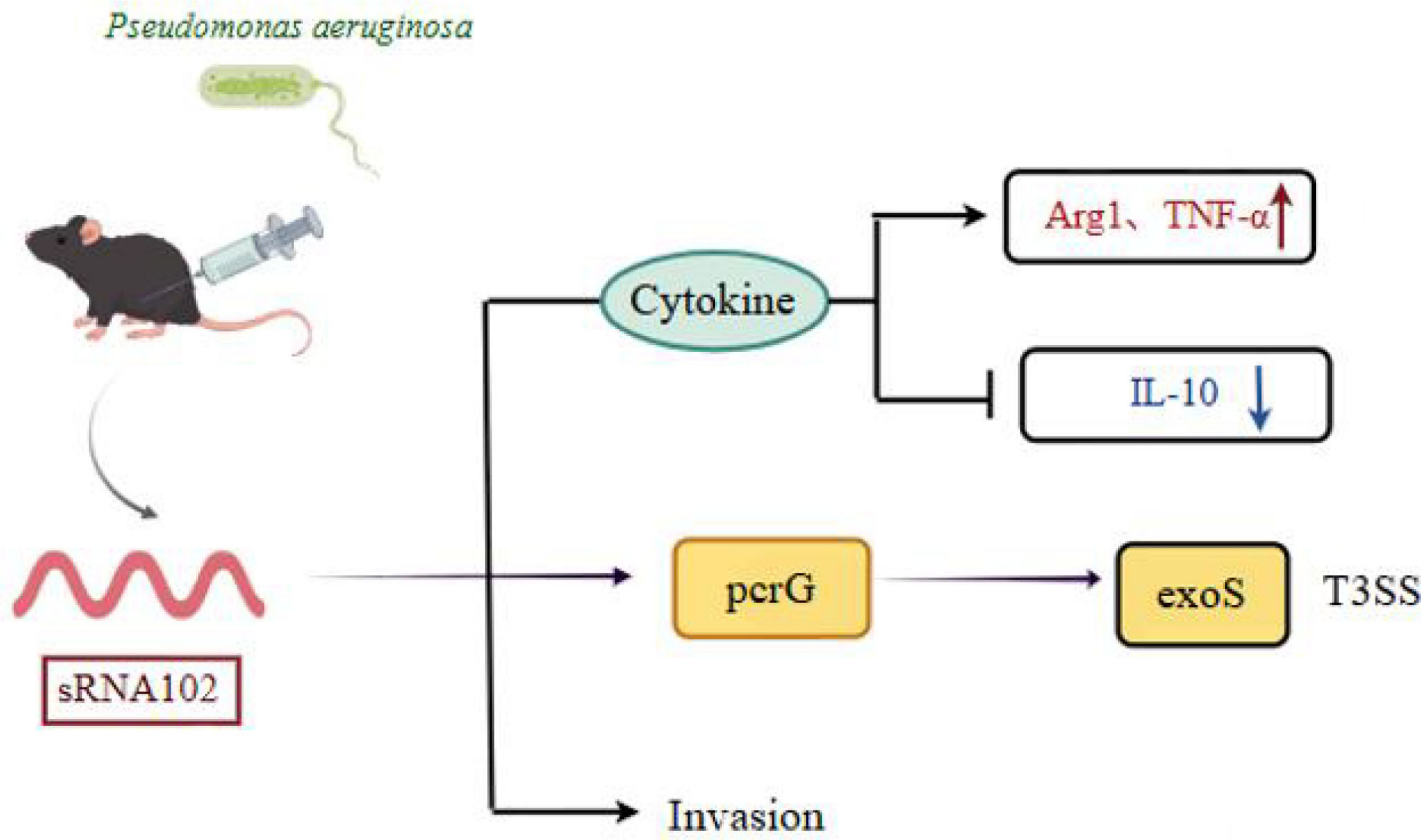
Schematic diagram of sRNA102 involvement in infection and immune regulation in *P. aeruginosa*.

This is the first study to demonstrate that sRNA102 regulates T3SS, interacts with the pcrG-exoS-T3SS pathway and the host immune regulatory network to modulate bacterial virulence and immune evasion (**Figure 5**). Collectively, these findings add to the current understanding of the sRNA effect on the bacterial environmental adaptation and uncovers important targets that can be leveraged to develop anti-infection strategies targeting the sRNA-axis to treat *P. aeruginosa* infections, such as sRNA inhibitors or pcrG antagonists (Wu et al., 2024).

## Data Availability

The original contributions presented in the study are included in the article/**Supplementary Material**. Further inquiries can be directed to the corresponding authors.
