# Supplementary material for: The small non-coding RNA sRNA102 regulates Pseudomonas aeruginosa virulence and host immunity by targeting the T3SS component PcrG
Source: Front Cell Infect Microbiol. 2026 Feb 11;16:1675468. doi: 10.3389/fcimb.2026.1675468 (PMC12932574; doi:10.3389/fcimb.2026.1675468)
Supplement: Supplementary file 1 [file Table1.docx]

**Supplementary Tables**

**Supplementary Table 1.** Sequence of primers.

| primer | sequence（5’ to 3’） | | |
| --- | --- | --- | --- |
| In28-*sRNA102*-F | | | TCCTCTAGAGTCGACCTGCAGGCGCGCCACATAGCTGGCATTGCCC |
| In28-*sRNA102*-R | | | ACGACGGCCAGTGCCAAGCTTCGAACAGGCATTGCAGCTGGGTTTG |
| In200-*sRNA102*-F | | | ATCGGCTCGTATAATGAATTCGCGCGCCACATAGCTG |
| In200-*sRNA102*-R | | | CGAATTTTAACAAAAGAATTCCGAACAGGCATTGCAG |
| In200-*pcrG*-F | | | ATCGGCTCGTATAATGAATTCTGAACGAATACACCGAAGACACCC |
| In200-*pcrG*-R | | | CGAATTTTAACAAAAGAATTCCCTTGCCACATTTCCGCCA |
| 30T-*pcrG*-F | | | GCTCTAGATGAACGAATACACCGAAGACACCC |
| 30T-*pcrG*-R | | | CATGCCATGGCCTTGCCACATTTCCGCCA |
| *rpod*-F | | | CTGAAGATCGCCAAAGAGCC |
| *rpod*-R | | | GTGTGGTCGGTGTTCATGTC |
| GAPDH-F | | | AGGAGCGAGACCCCACTAACA |
| GAPDH-R | | | AGGGGGGCTAAGCAGTTG |
| *sRNA102*-F | | | CACCGAACTTCTTGGCGA |
| *sRNA102*-R | | | CAAAAGCCCGAGGAGACC |
| *pcrG*-F | | | TGAACGAATACACCGAAGACACCC |
| *pcrG*-R | | | CCTTGCCACATTTCCGCCA |
| *exoS*-F | | | ATGCATATTCAATCGCTTCA |
| *exoS*-R | | | TCAGGCCAGATCAAGGC |
| *sRNA102*-P1 | | | ACGGCCAGTGAATTCGGGCCCCTCGCATACACGCGGCAT |
| *sRNA102*-P2 | | | TGGTCTGGTCCGGGAAGTGGTGAAGGAGAAG |
| *sRNA102*-P3 | | | CCTTCACCACTTC CCGGACCAGACCAGACCC |
| *sRNA102*-P4 | | | GGCTGGATCCCAAGCTCTAGATATGGCGCCCAATCGCTG |
| *sRNA102*-M1 | | | CACCGAACTTCTTGGCGA |
| *sRNA102*-M2 | | | CAAAAGCCCGAGGAGACC |
| Arg1-F | | | CAGAAGAATGGAAGAGTCAG |
| Arg1-R | | | CAGATATGCAGGGAGT |
| TNF-α-F | | | GCACCACCATCAAGGACTCA |
| TNF-α-R | | | AAGAGGAGGCAACAAGGTAGAG |
| IL-10-F | | | CTTACTGACTGGCATGAGGATCA |
| IL-10-R | | | GCAGCTCTAGGAGCATGTGG |

**Supplementary Table 2.** Sequence of probes.

| probe | sequence（5’ to 3’） |
| --- | --- |
| sRNA102 | GCUGGGUUUGAUCGACGGCCUGGGCAAUGCCAGCUAUGUGG |
| 5S | GUUUCACUUCUGAGUUCGGCAUGGGAUCAGGUGGG |
